# Determination of optimum dose based of biological responses of lethal dose (LD_25, 50, 75_) and growth reduction (GR_25, 50, 75_) in ‘Yaghouti’ grape due to gamma radiation

**DOI:** 10.1038/s41598-023-29896-z

**Published:** 2023-02-15

**Authors:** Ali Akbar Ghasemi-Soloklui, Mojtaba Kordrostami, Rouhollah Karimi

**Affiliations:** 1 Nuclear Agriculture Research School, Nuclear Science and Technology Research Institute (NSTRI), P.O. Box 31485498, Karaj, Iran; 2 Department of Landscape Engineering, Faculty of Agriculture, Malayer University, Malayer, 6571995863 Iran

**Keywords:** Plant breeding, Plant physiology

## Abstract

Ionizing radiations are a helpful technique and have improved financial potential in developing new and unique commercially important fruit tree varieties. The ‘Yaghouti’ grape cuttings were treated with 0 (control), 10, 20, 30, 40, 50, 60, 70, 80, 90, and 100 Gy gamma ray (γ) doses. The objectives of this study were to (1) investigate the effects of γ radiation on the survival rate, leaves, shoots, and root morphometric traits after γ irradiation; (2) Measurement of the 25, 50, and 75% lethal dose and 25, 50, and 75% growth reduction dose based on leaves, shoots, and root morphometric characteristics to estimate grape radiosensitivity; (3) Ultimately, determining the optimum dose of γ irradiation based biological responses (LD_25, 50, 75_ and GR_25, 50, 75_) in Yaghouti grape. The findings demonstrate that the lethal dose of the ‘Yaghouti’ was 18 Gy for LD_25_, 30 Gy for LD_50_, and 48 Gy for LD_75_, respectively. Furthermore, our findings showed that increasing the γ dose had a harmed ngative effect on vine growth, as evidenced by a decrease in plant height, root number, root volume, leaf area, aerial biomass, root biomass, and internode number of the ‘Yaghouti’ grape plants. Our results showed that between the aerial parts of vines, the leaf area and aerial biomass had higher radiosensitivity than plant height and other aerial parts of the plants based on data from GR_25_, GR_50_, and GR_75_. Moreover, GR studies of root characteristics revealed that root number and biomass root had higher radiation sensitivity than root volume. According to biological responses (LD_25, 50, 75_ and GR_25, 50, 75_) in the ‘Yaghouti’ grape, 30 Gy of γ radiation is the optimum dose for preliminary mutagenesis investigations.

## Introduction

According to historical documents^1–3^, the grape (*Vitis vinifera* L.) is the oldest cultivated fruit in the region south of the Caucasus, from the Black Sea to the Caspian region of Iran. The grape trees cultivated and grown in Iran are also very well-known, where the more remarkable diversity of cultivars can be found. Currently, the grape tree is one of the greatest extensively planted fruit trees. Moreover, to the latest current study on world viticulture by the Organization International de la Vigne et du Vin, grapes cover 7.4 million hectares of global surface area and produce 292 million hl^4^. In Iran, grape area cultivation is 158,000 hectares, with an annual production of 1,990,000 tons. As a result, viticulture is a prosperous agricultural sector in Iran; nonetheless, it demands specific cultivars with unique characteristics and qualities^5^. Increasing population, periodic food shortages, and the current and future effects of climate change have all led to an increased genetic resource for national and international food security. The demand for new cultivars that can be adapted to adverse areas is rising, which prompts the search for genetic materials^6^. The long juvenile stage, lack of acceptable collections, high height tree size, and other bridge barriers like incompatibility, stenospermocarpy, apomictic, and sterility are crucial challenges in fruit tree breeding. Thus, artificial mutations are beneficial in increasing natural diversity and have helped improve fruit yield and quality.

The ‘Yaghouti’ (synonymous with Shiraz) is a high-quality (i.e. rich in phenolic compounds, minerals, soluble sugars, and polyamines) seedless table grape cultivar produced in Iran. It has become an economically and socially important cultivar for producing early-maturing table grapes among vine-growers, especially in warm areas of Iran, such as Fars, Sistan-Baluchistan, and Kermanshah provinces, due to its remunerative price and high profitability. This cultivar also has a low chilling requirement, making it ideal for growing in subtropical areas with mild winters and short chilling hours, as well as grape-growing countries with dry and warm climates. The significant disadvantages of the Yaghouti are its highly compact bunches, cluster compactness, and small berry, which makes it difficult to wash the fruit before eating^7^. The number of seedless grape varieties in Iran is limited, and some have unfavorable characteristics; thus, superior seedless varieties must be bred.

Furthermore, the characteristics of seedlessness, lack of seed production, and spontaneous abortions in the Yaghouti cultivar have been expressed in previous research^8^. Consequently, breeding this cultivar by a conventional breeding method such as hybridization has proved challenging; therefore, mutation breeding can improve one or more undesirable traits of Yaghouti cultivar while maintaining its superior characteristic^9^. Moreover, unlike classical breeding methods, which generate new genetic variations from existing parental genes, the nuclear technique provides entirely new gene combinations with a high mutation rate^10^. Using ionizing radiation, such as protons, neutrons, α, β, X, and γ-rays, to induce mutation and generate genetics in fruit trees, is a primary instrument of nuclear technology for crop improvement. Thus, ionizing radiation may be helpful and have an improved financial potential in developing new and unique commercially important fruit tree varieties. γ-ray is commonly utilized in mutagenesis studies, because it has a shorter wavelength, penetrates deeper into the tissue, and has more energy per photon than X-rays^11^. On the other hand, γ-ray has proven to be incredibly useful, beneficial, and thriving in releasing the high number of mutant cultivars in the world^12^. Over the past six decades, several mutants of fruit tree species, including apple, citrus, sweet cherry, cherry, grape, peach, papaya, pear, and banana, have been introduced and are now widely employed in commercial orchards due to the use of γ irradiation^13^. The cumulative dosage and dose frequency are the most essential aspects of irradiation treatment, influencing mortality, reproductive, growth, and diversity of characteristics^14, 15^. Furthermore, irradiation treatments lead to a loss of pigments, total protein reduction, and vegetative growth prevention.

The primary metrics used to determine the appropriate ionizing radiation dosage to cause mutations in crop breeding are lethal dose (LD_50_) and growth reduction (GR_50_). Mutation induction causes high phenotypic variation and can be a result of genetic diversity that may possible to choose individual plants with beneficial traits that are not seen in nature. According to some researchers, the dosages where 50 percent of the total irradiated individuals die are those with the best likelihood of producing sustainable mutations for genetic improvement^16, 17^. In addition to the LD_50_, some studies indicate that the dose at which the 50% growth reduction (GR_50_) appears has a solid chance of causing successful mutants^18^. Furthermore, Thole et al.^19^ and Songsri et al.^20^ show that two metrics (LD_50_ and GR_50_) are predicated on the fact that low irradiation doses have a minor effect on the genome and seldom cause morphological variations. However, high irradiated doses could have many products on the genetic material and typically cause abnormalities or disadvantageous change. Thus, identifying the LD_50_ and GR_50_ is the preliminary stage in a mutation breeding method. Tetali et al.^21^ reported that the optimum dose for Indian grapes was 30 Gy, whereas another study found that the optimal dose for Indian cultivars was 15–25 Gy^20^, likewise the lethal doses of gamma rays for Turkish cultivars were 35 and 45 Gy^20^. Reviews of the literature indicate that there is a wide variation in the optimal dose among grape cultivars; as a result, these variations may be related to the genetic background of the cultivar. According to Alvarez-Holgun et al.^16^ the dose at which 50% of the irradiated samples die (LD_50_) or when 50% of plant growth has declined (GR_50_) increases the chance of producing viable and valuable mutants for the breeding program by using mutagenesis techniques. However, a new study demonstrated that the dose range below and above the LD _50_ (LD_25%_ and LD_75%_) and GR_50_ (GR_25%_ and GR_75%_) was critical for calculating the optimal dose whenever using γ irradiation^21^. Most earlier research on fruit trees specific grapes focused on determining the optimal dose by evaluating the survival rate (LD_50_) and morphological changes of the cutting's shoot and leaves in response to γ radiation dose. Thus, this is the primary study on determining the optimum dose of hardwood cuttings of grape under various γ radiation doses by focusing on GR_25, 50, 75_ and rather than LD_25, 50, 75_ based root, leaves, and shoot morphometric traits.

For this reason, the objectives of this study were: (1) investigate the effects of γ radiation on the survival rate, leaves, shoots, and root morphometric traits after γ irradiation; (2) Measurement of the 25, 50, and 75% lethal dose and 25, 50, and 75% growth reduction dose based on leave, shoot, and root morphometric characteristics to estimate grape radiosensitivity; (3) Ultimately, determining the optimum dose of γ irradiation based biological responses (LD_25, 50, 75_ and GR_25, 50, 75_) in Yaghouti grape. The results obtained from this study would be utilized in large-scale mutagenesis breeding programs or generating a wide range of mutants in grapes.

## Results

### Effect of mutagenesis on hardwood cutting survival percentage

Results of the Yaghouti grape reaction to γ irradiation treatments showed significant variations (*P* < 0.01) in survival rates between the 0 (control), 10, 20, 30, 40, 50, 60, 70, 80, 90, and 100 Gy treatments. The highest survival percentage (99.54%) was observed in the control, whereas the lowest survival percentage (18.36%) was recorded at 50 Gy (Table 1). Additionally, there was no cutting survival in the Yaghouti cultivar at 60 Gy and above (Table 1). Furthermore, the achievement has shown a progressive decline in the percentage survival with increased γ irradiation doses, except for doses of 10 Gy (79.02%) and 20 Gy (79.35%). Generally, hardwood cuttings had a greater chance of survival in lower and moderate doses of γ radiation treatment than in the highest doses (Table 1).

**Table 1:** The effect of different gamma irradiation doses on survival of hardwood cuttings of ‘Yaghouti’ grape (*Vitis vinifera* L.) cultivar.

Gamma dose (Gy)	Number of cutting	Survival (%)	Survival over control (%)	Reduction over control (%)
0	150	99.54	100	–
10	150	79.02	79.39	20.61
20	150	79.35	79.72	20.28
30	150	69.58	69.91	30.09
40	150	37.23	37.41	62.59
50	150	18.36	18.45	81.55
60	150	0	0	100
70	150	0	0	100
80	150	0	0	100
90	150	0	0	100
100	150	0	0	100

The means with same Duncan’s multiple range test letters in each column are not statistically significant (*P* ≤ 0.01). Data are average of four replications ± SE.

### Determination of LD_25_, LD_50,_ and LD_75_ of mutagens

**Figure 1:**
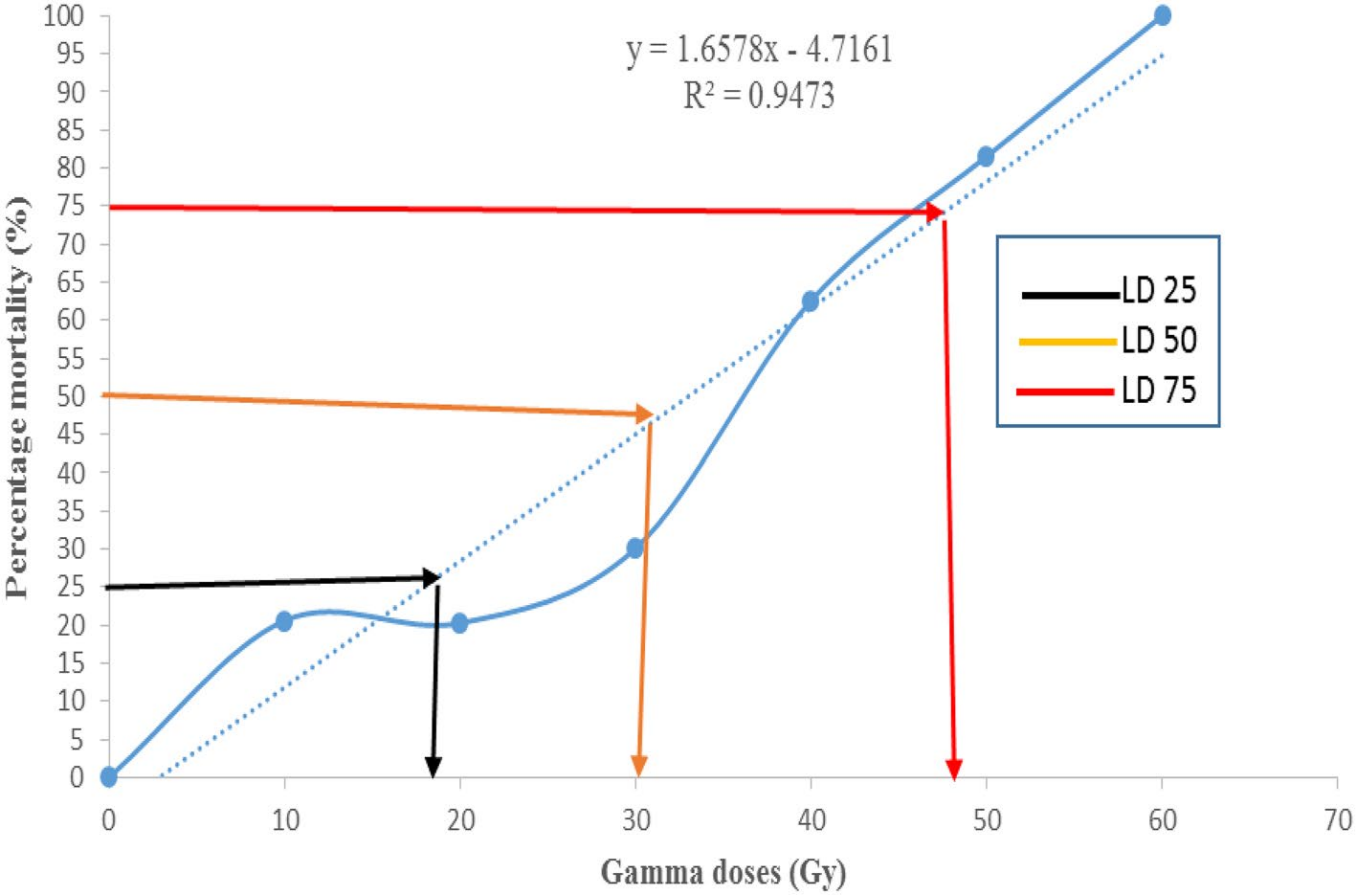
Analysis of Lethal dose (LD) on ‘Yaghouti’ grape (*Vitis vinifera* L.) cultivar irradiated with different doses of gamma-ray.

The linear regression equation model based on radiation doses is Y = 1.6578x–4.7161 and R2 equals 0.9473 (Fig. 1). According to the findings of this investigation, the lethal dose values for the Yaghouti cultivar were 18 Gy for LD_25_, 30 Gy for LD_50_, and 48 Gy for LD_75_ (Fig. 1). Thus, the Yaghouti cultivar can generate viable mutations in response to γ radiation dosages between 10 and 50 Gy; however, for preliminary mutagenesis investigations, 30 Gy seems to be the optimum dosage.

### Effects of mutagens on leaf traits

Effects of γ radiation on the M1 generation of the Yaghouti leaf characteristics are shown in Table 2. After γ radiation treatments, the Yaghouti grape leaf area ranged from 4475 mm^2^ (0 Gy) to 1452 mm^2^ (50 Gy). At 10–50 Gy, the leaf area declined to about 30% (10 Gy), 58% (20 Gy), 60% (30 Gy), 58% (40 Gy), and 67% (50 Gy) as compared to the control, although there were no significant differences at 20, 30, 40, and 50 Gy. Due to γ irradiation showed a considerably significant difference in leaf width and length in grapes (Table 2). The maximum leaf length (80.79 mm) and width (77.53 mm) were seen in the control, but the minimum leaf width (45.60 mm) and length (45.83 mm) were observed in the 50 Gy (Table 2). Furthermore, the leaf length and width of the Yaghouti cultivar gradually decreased as dose levels rose, but there were no significant differences between high dose γ irradiation (30, 40, and 50 Gy). As a result of the γ-ray, the fresh-weight leaves of cv. Yaghouti significantly decreased compared to the control, whereas no significant differences were observed between 10 Gy and the control in the dry weight of leaves. All gamma radiation doses significantly reduced the fresh weight of the cv. Yaghouti leaves compared to the control, although there were no significant differences between 10 Gy and control in values of the dry matter of the leaves (Table 2).

**Table 2:** The effects of different gamma irradiation doses on leaves morphometric traits of ‘Yaghouti’ grape (*Vitis vinifera* L.) cultivar.

Gamma dose (Gy)	Leaf area (mm^2^)	Leaf width (mm)	Leaf length (mm)	Fresh weight (g)	Dry weight (g)
0	4475.67 ± 352 a	80.70 ± 2.90 a	77.53 ± 3.81 a	0.75 ± 0.02 a	0.15 ± 0.009 a
10	3133.00 ± 16 b	61.83 ± 1.29 b	63.33 ± 1.36 b	0.57 ± 0.005 b	0.12 ± 0.004 a
20	1853.67 ± 141 c	51.47 ± 1.98 c	62.03 ± 2.06 b	0.41 ± 0.02 c	0.07 ± 0.008 b
30	1782.67 ± 15 c	50.30 ± 0.131 c	50.87 ± 0.38 c	0.37 ± 0.03 c	0.05 ± 0.006 b
40	1876.00 ± 66 c	50.63 ± 0.80 c	50.60 ± 0.30 c	0.35 ± 0.002 c	0.07 ± 0.001 b
50	1452.67 ± 80 c	45.60 ± 0.65 c	45.83 ± 2.12 c	0.37 ± 0.02 c	0.09 ± 0.008 b

The means with same Duncan’s multiple range test letters in each column are not statistically significant (*P* ≤ 0.01). Data are average of four replications ± SE.

### Effects of mutagens on shoot traits

**Figure 2:**
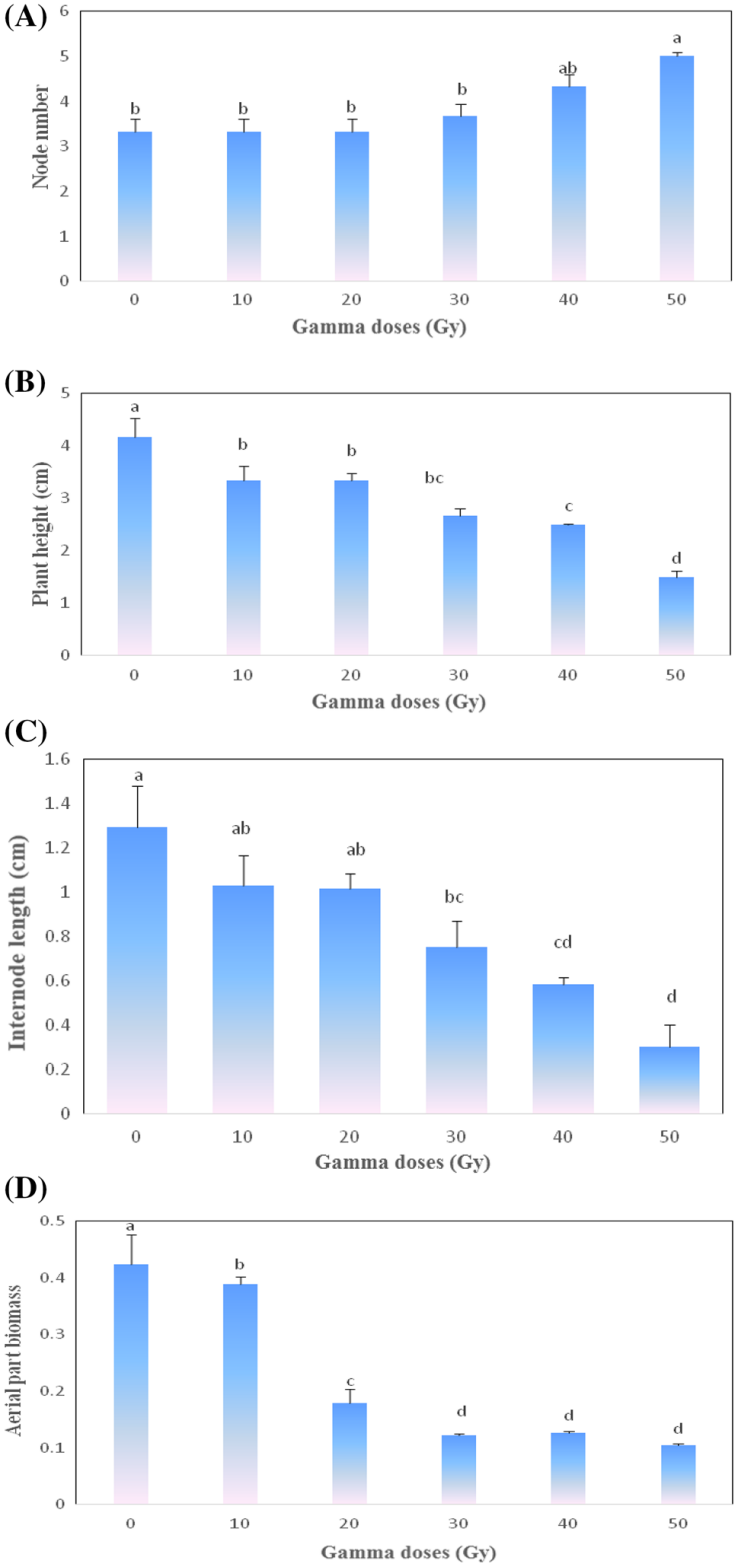
The effect of different gamma irradiation doses on shoot morphometric traits (nod number, panel **A**; plant height, panel **B**; internode length, panel **C**, aerial part biomass, panel **D**) of ‘Yaghouti’ grape (*Vitis vinifera* L.) cultivar. The means with same Duncan’s multiple range test letters in each column are not statistically significant (*P* ≤ 0.01). Data are average of four replications ± SE.

There were significant differences between the control and γ-irradiated cutting (doses of 10 to 100 Gy) for plant height and aerial parts biomass. However, for node number and internode distance, there were no significant differences between the control and lower doses of γ-ray irradiations (Fig. 2). Under various irradiation doses, the mean values for node number and internode length ranged from 3.33 to 5 and 1.29–0.3 cm, respectively. Moreover, the rise in radiation intensity was defined as a significant reduction in the mean values of the shoot, as well as the maximum decrease in node number and internode length related to 40 and 50 Gy compared to other treatments. According to the results, increasing the dose of γ irradiation decreased the plant height of the Yaghouti cultivar by 64% in 50 Gy compared to the control treatment. In comparison to the control (4.16 cm), the lowest plant heights were related to 50 Gy (1.5 cm), 40 Gy (2.5 cm), 30 Gy (2.6 cm), and 20 Gy (3.3 cm), respectively (Fig. 2).

The lowest plant biomass was observed in grapevines treated with 30, 40, and 50 Gy; while the highest biomass was observed in 10 Gy (Fig. 2). Moreover, the control (0 Gy) plant showed the highest biomass accumulation (up to three-fold) than 50 Gy (Fig. 2).

### Effects of mutagens on root traits

Similar trends were seen for root characteristics, except for the highest root, which reduced gradually as γ irradiation doses increased from 10 to 50 Gy, as shown in Table 3. On the other hand, non-irradiated treatment (control) and a dose of 10 Gy had no significant differences in their effects on the number of the roots. In contrast, high doses decreased the root number from 41.33 in control to 9 in 40 Gy. Compared to non-irradiated treatment, a cutting exposed to 20 and 30 Gy produced an intermediate number of roots (30–19.6) (Table 3).

**Table 3:** The effect of different gamma irradiation doses on root traits in ‘Yaghouti’ grape (*Vitis vinifera* L.) cultivar.

Gamma dose (Gy)	Root number	Root highest (cm)	Root volume (cm^3^)	Root fresh weight (g)	Root biomass (g)
0	41.33 ± 1.09 a	22.00 ± 0.94 b	4.83 ± 0.14 a	3.30 ± 0.55 a	0.25 ± 0.03 a
10	39.33 ± 1.19 a	20.00 ± 1.89 b	3.75 ± 0.12 b	3.13 ± 0.23 ab	0.20 ± 0.02 ab
20	30.33 ± 1.36 b	24.67 ± 0.54 b	3.33 ± 0.27 bc	2.18 ± 0.16 bc	0.16 ± 0.01 abc
30	19.67 ± 2.60 c	24.00 ± 2.49 b	2.75 ± 0.12 cd	1.52 ± 0.22 c	0.14 ± 0.04 bc
40	9.33 ± 0.98 d	25.33 ± 2.13 b	2.50 ± 0.41 cd	1.27 ± 0.14 c	0.16 ± 0.02 abc
50	10.00 ± 0.47 d	32.33 ± 1.19 a	2.33 ± 0.27 d	1.11 ± 0.001 c	0.07 ± 0.001 c

The means with same Duncan’s multiple range test letters in each column are not statistically significant (*P* ≤ 0.01). Data are average of four replications ± SE.

The results from Table 2 show that low and moderate γ doses do not influence root elongation, whereas increasing the γ dose to 50 Gy caused root elongation. Furthermore, there were no significant differences between cuttings that had received 10–40 Gy of radiation. Cuttings exposed to 50 Gy of radiation showed the highest elongation root (32.33 cm); however, explants exposed to 0–10 Gy of radiation showed the shortest elongation root (20–22 cm). The volume of roots significantly decreased with increasing γ-ray doses from 10 to 50 Gy (Table 3). Hardwood cutting irradiated with 20–50 Gy exhibited fewer volume roots than non-irradiated cutting; otherwise, no significant differences were observed between cutting irradiated with 10 Gy and non-irradiated. Furthermore, higher dose (50 Gy) γ radiation has a significant impact on root volume reduction compared to low dose γ radiation. Additionally, fresh root weight and biomass decreased with an increase in radiation exposure above 20 Gy (Table 3). The fresh weight of the root was reduced by 50–75% in 30 and 50 Gy, but there were no significant differences between 10 Gy γ irradiation and non-irradiated treatment (Table 3). In contrast to the control, the lowest root biomass was observed after exposure to 30 and 50 Gy of γ radiation. Furthermore, lower doses (10 and 20 Gy) were similar to the control treatment, whereas 30 and 50 Gy had the most effect on the reduction of root biomass (Table 3).

### The radiosensitivity test

**Figure 3:**
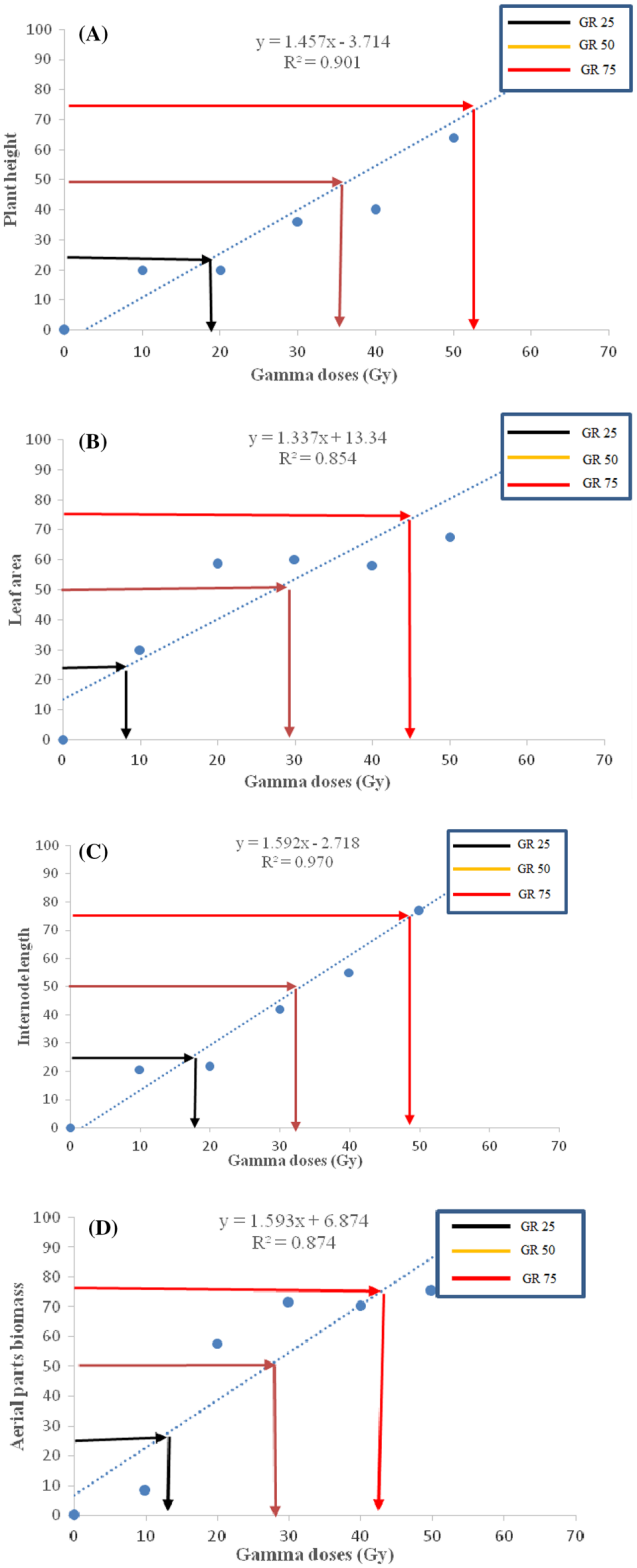
The effect of different gamma irradiation doses on growth reduction of shoot and leaves morphometric traits (plant height, panel **A**; leaf area, panel **B**; internode length, panel** C**, aerial part biomass, panel **D**) of hardwood cutting of ‘Yaghouti’ grape (*Vitis vinifera* L.) cultivar.

**Figure 4:**
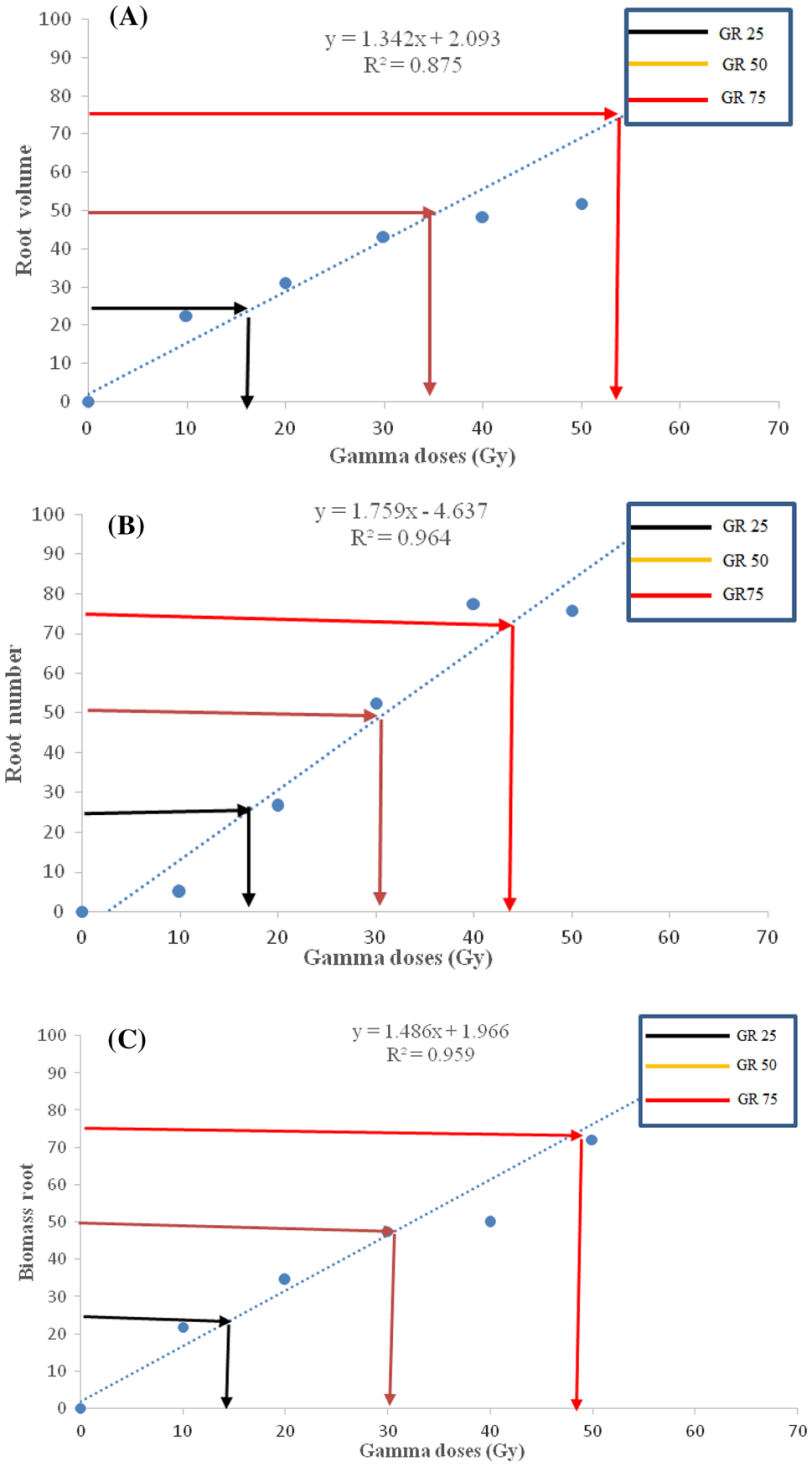
The negative effect of different gamma irradiation doses on growth reduction of root morphometric traits (root volume, panel **A**; root number, panel **B**; biomass root, panel; **C**) of hardwood cutting of ‘Yaghouti’ grape (*Vitis vinifera* L.) cultivar.

**Figure 5:**
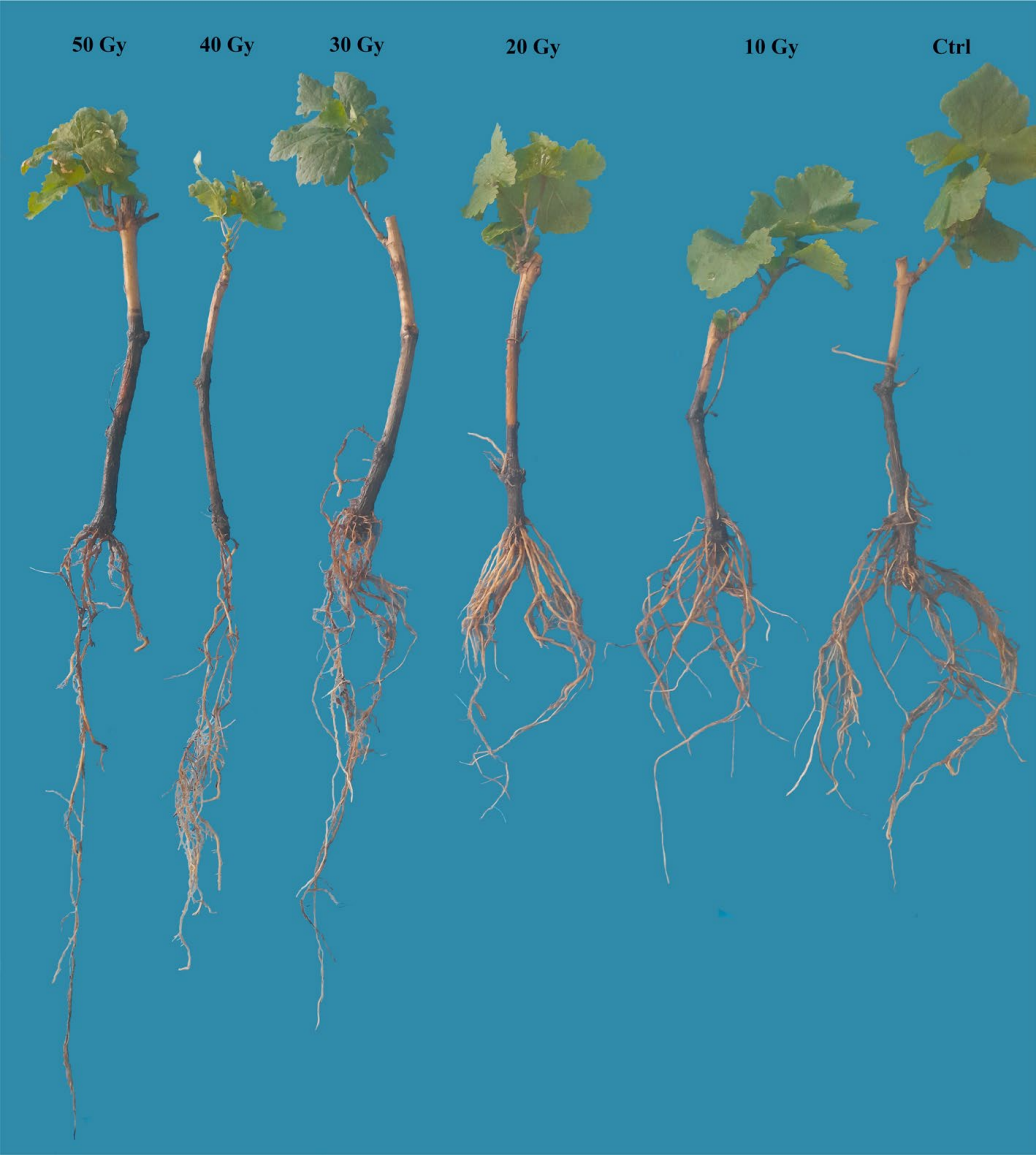
The negative effect of different gamma irradiation doses on morphometric traits of hardwood cutting of ‘Yaghouti’ grape (*Vitis vinifera* L.) cultivar.

The growth reduction (GR) in γ-irradiated grape cutting from this study was determined using the resulting regression equation. Also, the grape growth reduction was calculated for 25% (GR_25_), 50% (GR_50_), and 75% (GR_75_) (Figs. 3 and 4). Our findings showed that increasing the γ dose harmed grape growth, as evidenced by a decrease in plant height, root number, root volume, leaf area, aerial biomass, root biomass, and internode number of the ‘Yaghouti’ grape (Fig. 5). Moreover, the regression equation's determination coefficient (R^2^) ranged from 0.87 to 0.96 for root characteristics. In contrast, it varied from 0.85 to 0.97 for traits related to leaves and shoots (Figs. 3 and 4).

The GR_25_ values for leaf area, biomass shoot, internode length, and plant height, were 9, 12, 17, and 19 Gy, respectively (Fig. 3). At the same time, the GR_50_ for plant height was 35 Gy, for internode length was 32, and for aerial biomass and leaf area were 29 Gy. In the meantime, the GR_75_ value for all characteristics of the aerial parts in grapes was 42–52 Gy. Our results showed that the leaf area and aerial biomass had higher radiosensitivity than the aerial parts of the grape, based on data from GR_25_, GR_50_, and GR_75_ (Fig. 3).

GR_25_ values for root morphometrically traits such as root number, root volume, and biomass root in grape cutting were 15–18 Gy. In contrast, the GR_50_ values for root number, root biomass, and root volume were 30, 30, and 35 Gy, respectively. GR_75_ for root characteristics showed a more comprehensive range (45–53 Gy) than GR_25_ and GR_50_ values, whereas the highest value of GR_75_ related to root volume (53 Gy), biomass root (49 Gy), and root number (44 Gy). Therefore, GR studies of root characteristics revealed that root number and biomass root had higher radiation sensitivity than root volume. The results showed that various plant tissues have different degree of sensitivity to γ radiation.

### Multivariate analyses

**Figure 6:**
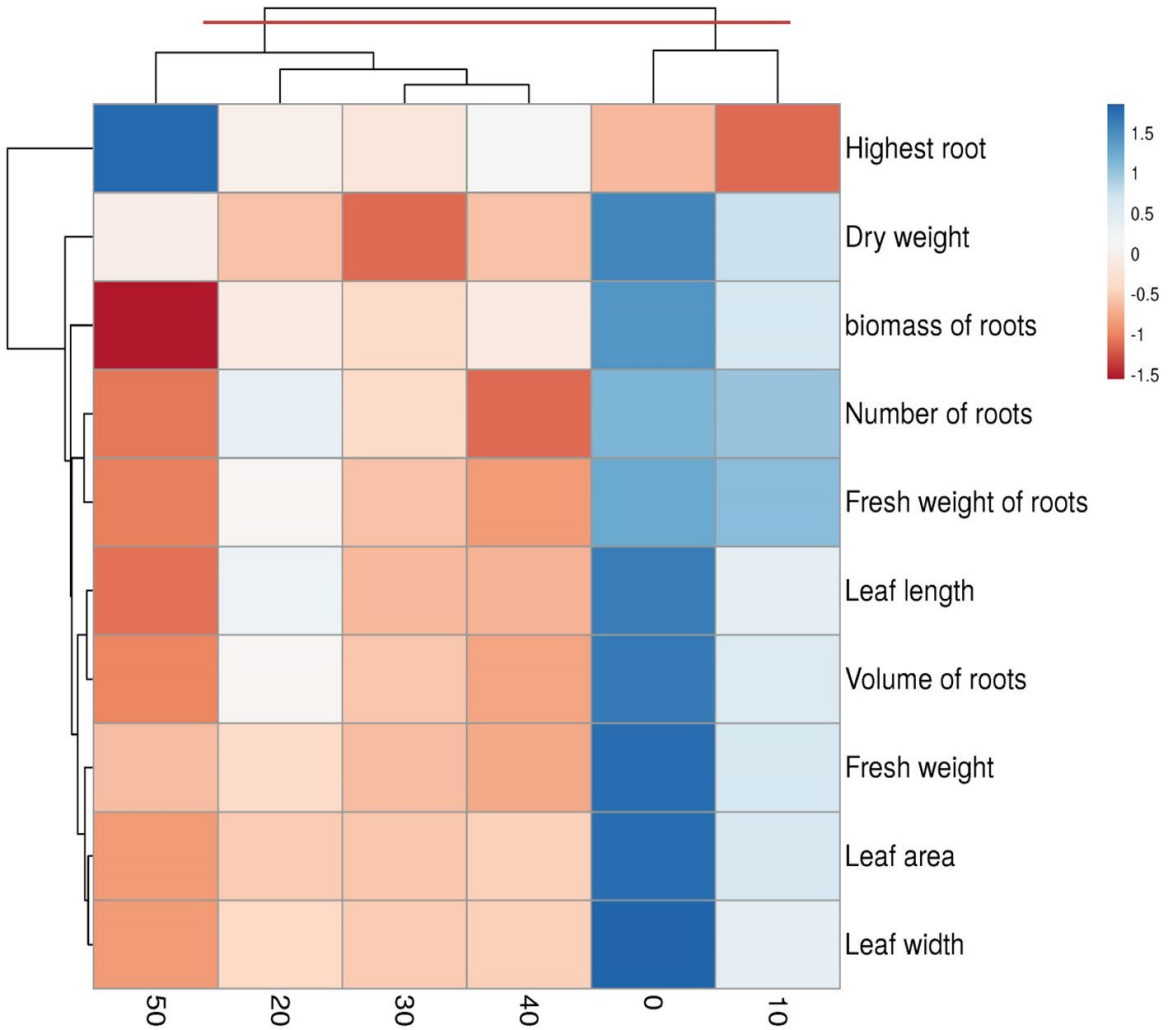
Cluster analysis of the studied traits in different gamma treatments.

To better understand the behavior of traits under different γ-ray treatments, we used cluster analysis (Fig. 6). Based on this, two groups were formed. In the first group, the control and 10 Gy treatments were included. The control treatment was superior to other treatments in terms of most traits. It shows the effect of γ-ray on the studied morphological characteristics well. Also, in this group, the control treatment was superior to the 10 Gy treatment. However, these treatments were better than other treatments. But the second group had two subgroups. The first subgroup had three treatments of 20, 30, and 40 Gy. Among the treatments of this group, only the 40 Gy had the minimum value in terms of most attributes.

**Figure 7:**
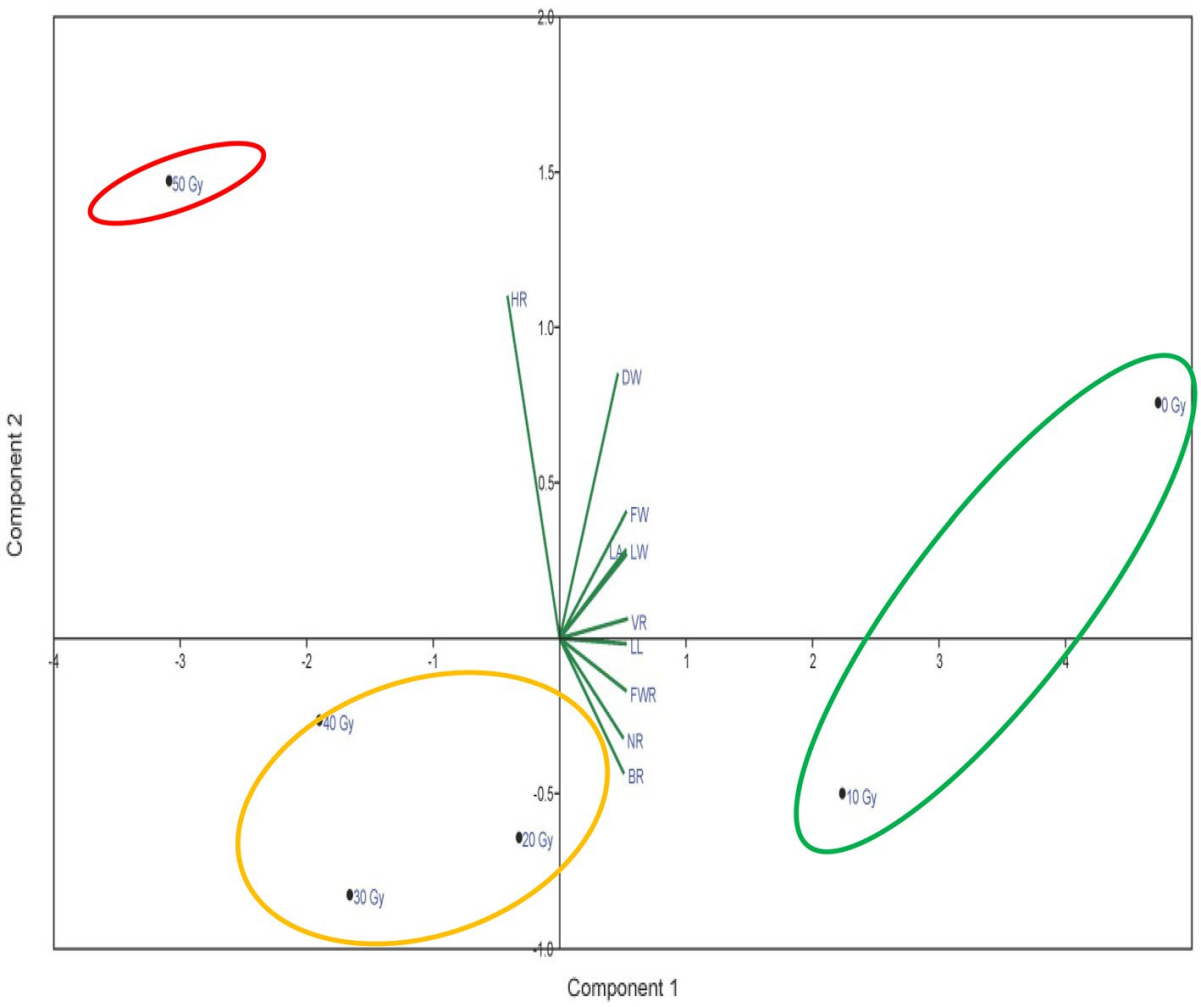
Principal component analysis (PCA) of the studied traits in different gamma treatments. Highest root (HR); Dry weight (DW); Biomass of root (BR); Number of root (NM); fresh weight of root (FWR); Leaf length (LL); Volume of root (VR); Fresh weight (FW); Leaf area (LA) and Leaf width (LW).

Nevertheless, the 30 Gy had a better condition than the other treatments. This treatment was weaker than the 20 Gy treatment in dry weight. There was only one treatment in the second subgroup. The 50 Gy was fragile compared to other γ radiation. It shows the reducing effect of a high dose of γ radiation on most of the morphological traits in this study. It is interesting to note that the principal components analysis results confirmed the cluster analysis (Fig. 7). Based on this, three groups were formed. In the first group, the control and 10 Gy treatments were included; these treatments had very high values in terms of most traits. The second category had 20, 30, and 40 Gy. Among the treatments of this group, only the 40 Gy had the minimum value for most attributes. However, the 30 Gy had a better condition than the other treatments, while the 50 Gy was fragile irradiation for the Yaghouti cultivar.

## Discussion

Results of the Yaghouti grape reaction to γ irradiation treatments showed that hardwood cuttings had a greater chance of survival in lower and moderate doses of γ radiation than in the highest doses. The pattern of reduced survival rate with increasing γ dose is comparable to previous mutagenesis studies in grapes by Surakshitha and Soorianathasundaram^22^; in strawberries by El Oualkadi et al.^23^; in citrus by Sutarto et al.^24^. At higher doses the survival rate could well have decreased due to chromosomal damage, suppression of critical cell functions, or a combination of both^25, 26^, which finally led to bud death and prevented bud break in cutting^24, 25^. Moreover, the cv. Yaghouti hardwood cuttings exposed to doses of 60 and above γ radiation exhibited a 100% mortality rate. Contrasting to these observations, Surakshitha and Soorianathasundaram^22^ showed that 30 Gy γ irradiation caused a 100% death rate in Indian grape varieties such as ‘Red Globe’ and ‘Muscat’.

According to the findings of this investigation, the lethal dose values for the Yaghouti cultivar were 18 Gy for LD_25_, 30 Gy for LD_50_, and 48 Gy for LD_75_, respectively. Determining the perfect radiation dose (LD_50_), or the amount that eliminates 50 percent of the total population is necessary for the efficient induction of mutagenesis by ionizing radiation. Similarly, Sharma and Mukherjee^27^ indicated that the effective dose for inducing mutations in the ‘Pusa Seedless’ grape is 30 Gy. According to the mortality rate of grape cuttings, Coban^28^ optimized the LD_50_ for the grapes cv. ‘Mevlana’, ‘Pembe Gemre’, and ‘Misket’ at 38 Gy. Other authors suggest that the LD_50_ dose of γ-ray for the grape cultivars ‘Red Globe’ and ‘Muscat’ from the Indian cultivar is 15–20 Gy, whereas the LD_50_ dose for the grape cultivar ‘Black Matrouh’ from the Egyptian cultivar was discovered to be 50 Gy. These different LD_50_ doses for grape cultivars may be due to the variations of moisture content in cutting and the genetic diversity of grape cultivars. Because the cytoplasm of plant cell contains about 80% water, thus biological effect of γ-ray depend interaction with water molecules to produce free radicals and plant cell, which can diffuse far enough to reach and damage different important components in vegetative cells^29^.

Leaf area is a major index in many physiological and plant modeling studies, thus evolution of leaf area index as a fast and non-destructive technique requirement to each plant breeding study^29^. The highest leaf area values were obtained from the control and 15 Gy Co^60^ treatments. Ekbiç et al.^9^ reported that progressive radiation doses caused a variation in leaf area values in grapes. Moreover, Sparrow^30^, who studied the cytological effects of radiation, concluded that these cytological alterations, including chromosomal damage, slowed mitosis, nuclear deterioration, cell expansion, and others, cause the reduction in vegetative traits such as leaf area. Nee Tripathi and Kumar^31^ reported that higher doses of γ-ray lead to various drastic physiological effects, including changed protein biosynthesis, change in water transport, modified leave gas exchange, and hormones and enzymes imbalance. Moreover, Kovacs and Keresztes^32^ reported that continuous short-wave radiation exposure would restrict vegetative development due to the gradual leaf elongation rate, resulting in lesser fully expanded leaves. Similar results were reported by Surakshitha and Soorianathasundaram^22^, who observed decreasing in leaf length, and leaf width by γ-ray treatment. Some of the traits leave including fresh and dry weight, leaf length, and width of a leaf revealed a decreasing pattern with a rise in γ radiation. These findings are consistent with El Sherif et al.^33^, who showed that γ-ray exposure significantly reduced the fresh and dry weight of rosell leaves than control.

Moreover, our data showed that the plant height and biomass grape were reduced by γ irradiation. The main biological effect of irradiation mutagen was a delay or reduction in cutting sprouting, a decrease in plant height, and a diversity of shoot traits in the early stages of plant growth^34^. Kiani et al.^35^ reported high doses of γ-ray treatment were caused decreeing of morphological characteristics such as stem length and stem weight in wheat. Furthermore, there were no significant differences in node number and internode distance between the control and low doses of γ irradiation, whereas high doses of γ irradiation resulted in dwarf plants with short internode distances. Additionally, Abdullah et al.^36^ reported that the variation in plant height in the mutants was caused by an alteration in the intermodal length. Growth internode decreases may be due to auxin disintegration, ineffective assimilation systems, or suppression of mitosis and chromosomal destruction, leading to secondary physiological and morphological effects. After treated with a high dose of γ radiation grape growth was led to a reduction in height, node number, and internode length. Similar findings were reported by Tayyar et al.^37^ in grape cv. Amasya; Surakshitha and Soorianathasundaram^22^ in grapes cv. Red Globe and Muscat; Yadav^38^ in *Canscora decurrens* Dalz; Al-Mousa et al.^36^ in grape cv. Black Matrouh.

On the other hand, non-irradiated treatment (control) and 10 Gy γ-ray had no significant differences in their effects on the root number. In contrast, high doses decreased the root number from 41.33 in control to 9 (40 Gy) to 10 (50 Gy). In addition, a cutting exposed to 20 and 30 Gy of radiation produced an intermediate number of roots (30–19.6). Like our findings, Al-Mousa et al.^39^ showed that γ radiation in grape explants at a higher dose (40 Gy) markedly reduced root induction compared to non-irradiated. Additionally, they stated that non-irradiated treatment and explants that had received 10 and 20 Gy of radiation had the highest numbers of roots. Cuttings exposed to 50 Gy of radiation showed the highest elongation root (32.33 cm); however, explants exposed to 0–10 Gy of radiation showed the shortest elongation root (20–22 cm). Ali et al.^40^ found that tea cutting treated with 60 Gy γ radiation resulted in increased root length, whereas lowest root length observed in control plant. In contrast to our findings, Al-Mousa et al.^39^ revealed that 40 Gy-irradiated grape was caused to shortest roots. According to Hong et al.^41^, root length were marginally shorter under γ exposure than controls. Our findings contrast with those of Gaul^42^, who stated that rising radiation doses negatively impacted the M1 generation due to a decrease in root height in grapes. Moreover, El Sherif et al.^33^, the roselle plant's root length was significantly longer after γ irradiation compared to the control. Also, Thapa^43^ observed that γ-ray treatments at an initial stage inhibited root and hypocotyl extension in two pines species. These different results in root trait of plant to γ-ray treatments may be due to the variations of genetic diversity of plants and differences of plant material used.

Radiosensitivity experiments can estimate radiation doses to cause the highest rate of mutations with minimal effect on the gene complex^44^. Determining the effective radiation dosage, also known as the dose that decreases survival by 50% (LD_50_) or population growth by 50% (GR_50_), is necessary for the efficient induction of mutagenesis by γ radiation. Thus, both parameters depend on factors such as moisture content, stage of growth development, and plant tissue^17, 45^. Our findings demonstrated GR_50_ for the shoot, leave, and root in the ‘Yaghouti’ grape varied from 29 to 35 Gy. According to Tayyar et al.^37^, the ‘Amasya’ grape had a GR_50_ value of 21.46 Gy, whereas Pannuswami et al.^46^ discovered that the GR_50_ dose in the Muscat grape was 20–25 Gy. It was stated that the ‘Yaghouti’ is comparatively radiation-resistant and would be helpful for phytoremediation by radionuclides.

## Conclusion

According to the findings of this investigation, the lethal dose values for the ‘Yaghouti’ were 18 Gy for LD_25_, 30 Gy for LD_50_, and 48 Gy for LD_75_. The leaf area declined with an increase in the irradiation dose. As a result of the γ-ray, the fresh weight leaves of cv. Yaghouti significantly decreased compared to the control. Also, the rise in radiation intensity was defined as a considerable reduction in node number and internode length. On the other hand, non-irradiated treatment and 10 Gy γ-rays had no significant differences in their effects on the root number; while, high doses of γ-rays decreased the root number from 41.33 in control to 10 (50 Gy). Moreover, GR studies of root characteristics revealed that root number and biomass root had higher radiation sensitivity than root volume. The results made it evident that various plant parts had different levels of radiosensitivity. Our results showed that the leaf area and aerial biomass had higher radiosensitivity than the aerial parts of the grape. According to biological responses (LD_25, 50, 75_ and GR_25, 50, 75_) in the ‘Yaghouti’ grape, 30 Gy of γ radiation is the optimum dose for preliminary mutagenesis investigations.

## Materials and method

### Plant material

The ‘Yaghouti’ grape cultivar (*Vitis vinifera* L.) was used in this study. On December 2, 2021, a uniform size one-year-old cutting of the "Yaghouti" grape from ten-year-old trees was collected from the commercial orchard of Kavar City in the southern province of Fars, Iran. Grape cuttings (15–20 cm length and 0.5–1.0 cm diameter) were sterilized by bathing them in a solution of 4000 ppm benomyl (Ariashimi Co., Zahedan, Iran) for 5 min before being wrapped in wet paper, packed in plastic bags, and kept at 5 °C for transportation from the field to the laboratory.

### Gamma irradiation

Eleven groups of grape cuttings were prepared. The first group was maintained as the control and other samples were treated with 10, 20, 30, 40, 50, 60, 70, 80, 90, and 100 Gy γ-irradiation doses at the Nuclear Science and Technology Research Institute in Karaj, Iran. A total of 1500 cuttings, excluding the control, were exposed to γ radiation through fifty cuttings with double bud canes for each dose and replication. Both control and γ irradiated cuttings were deep in indole- 3-butyric acid (3000 ppm in 50% ethanol) for five seconds (quick dip technique), and planted in 10-lit plastic bags containing sand. During early December until cuttings begin to root in a controlled greenhouse with 24 °C/18 °C day/night temperatures and 60% RH. The rooted cuttings were irrigated every three days and supplied twice a week with 0.5 L half-strength Hoagland's solution for 16 weeks^5^.

### Measurements of plant growth characters

The number of dead cuttings divided by the total number of treated cuttings for each irradiation dose was used to calculate the survival percentage. Four months after planting and under optimal conditions, measurements were made of the plant height, internode length, number of nods, leaf width, fresh weight, and dry weight of leaves. The total leaf area was calculated using Image J software and an automatic electronic leaf area meter (model LI-3000, U.S.A.). Additionally, measurements of root morphometric traits such as the number of roots, highest root, and root volume were made. After separating the roots from the base of the cuttings, a digital balance was used to assess the fresh weight. The samples of shoots, leave, and roots were dried in an oven for 72 h at 65 °C and then the samples were weighed using a Mettler Viper BC analytical scale after dried for measurement of the dry weight of shoot, dry weight of shoot, root biomass and shoot biomass. Furthermore, the root volume was calculated using a graduated cylindrical.

The mean lethal dose (LD_25_, LD_50,_ and LD_75_) was estimated using the resulting regression equation from the survival percentage. The same procedure was utilized to estimate the mean growth reduction (GR_25_, GR_50_, and GR_75_) for shoot, root, and leaf.

### Statistical analysis

The experiment design was in the form of a completely randomized design with four replications. The statistical significance of the differences between the mean values was determined using a one-way analysis of variance with Duncan’s multiple-range tests. Significant differences were evaluated at a 1% level of significance.

### Ethical approval

We confirm that all the experimental research and field studies on plants (either cultivated or wild), including the collection of plant material, complied with relevant institutional, national, and international guidelines and legislation. All of the material is owned by the authors, and or no permissions are required.

## Data Availability

Some or all data, models, or codes that support the findings of this study are available from the corresponding author upon reasonable request.
